# Effects of Aspen Wood Torrefaction Condensate Addition on *Porphyridium marinum* Growth, Biomass Composition, and Exopolysaccharide Production

**DOI:** 10.3390/md24070253

**Published:** 2026-07-20

**Authors:** Salini Chandrasekharan Nair, Amal D. Premarathna, Kārlis Dieviņš, Christine Gardarin, Marju Robal, Céline Laroche, Rando Tuvikene, Renu Geetha Bai, Timo Kikas

**Affiliations:** 1 Chair of Biosystems Engineering, Institute of Forestry and Engineering, Estonian University of Life Sciences, Kreutzwaldi 56, 51014 Tartu, Estonia; salini.chandrasekharan@emu.ee; 2 School of Natural Sciences and Health, Tallinn University, Narva mnt 29, 10120 Tallinn, Estonia; amald@tlu.ee (A.D.P.); marju.robal@gmail.com (M.R.); rantuv@tlu.ee (R.T.); 3 Institute of Bioengineering, Faculty of Science and Technology, University of Tartu, Nooruse 1, 50411 Tartu, Estonia; karlisdievins@gmail.com; 4 Université Clermont Auvergne, Institut Pascal, UMR CNRS 6602, F-63000 Clermont-Ferrand, France; christine.gardarin@uca.fr (C.G.); celine.laroche@uca.fr (C.L.)

**Keywords:** amino acids, antioxidant assays, circular bio economy, fatty acids, microalgae, *Porphyridium* spp., thermochemical conversion process, wood biomass torrefaction

## Abstract

Torrefaction of biomass produces torrefied biomass, non-condensable gases and condensable gases, which can be cooled to form a torrefaction condensate (TC). It contains assimilated organic carbon and compounds capable of inhibiting microbial growth. This study evaluated whether TC produced from aspen wood chips torrefied at 225 °C could be incorporated into cultures of the red microalga *Porphyridium marinum* and how TC exposure affected growth, biomass composition, and exopolysaccharide (EPS) production. TC was added to established cultures at 0.5–2.5 mL/L. Harvested biomass and purified EPS were characterised by chromatographic, colorimetric, and antioxidant assays. TC caused immediate concentration-dependent growth inhibition followed by partial recovery. However, purified EPS yield was lower than the control (202.7 mg/L) in all TC treatments (118.4–185.5 mg/L). The biomass lipid fraction reached 47.47% at 0.5 mL/L, compared to 15.47% in the control, and decreased as TC dosage increased. EPS protein and sulphate contents were in the ranges of 2.08–2.89% and 8.54–9.56%, respectively, compared to 1.67% and 8.80% in the control. FTIR spectra indicated the preservation of principal EPS functional groups, whereas antioxidant activity was generally weak and assay-dependent. These findings demonstrate the tolerance and compositional acclimation of *P. marinum* to low aspen–TC loadings, but not improved biomass or EPS productivity. This study suggests a new route for integrating thermochemical conversions with cultivation of red microalgae; however, it requires detailed detoxification, process optimisation, and further investigation before biorefinery implementation.

## 1. Introduction

Thermochemical pre-treatment processes such as torrefaction convert biomass into energy-dense solid fuels while generating by-products such as condensable and non-condensable gases. Rather than directly releasing these into the atmosphere, the condensable gases containing valuable volatiles can be cooled to form a viscous liquid, called torrefaction condensate (TC). TC remains underutilised due to its complex composition [1,2], including organic acids, furfurals, phenolic compounds and other oxygenated organics. Current valorisation strategies primarily focus on recovering these compounds through separation or converting TC into value-added products such as volatile fatty acids and biomethane via anaerobic digestion [3,4]. However, the wide range of molecular weights and the polarities of heterogeneous TC components make their separation and recovery technically and economically challenging [4]. In addition to that, high organic acid and phenolic content exhibits inhibitory effects on microbial metabolism, limiting the efficiency of microbial degradation and conversion processes [1,5]. Due to these reasons, TC currently has limited applications, such as fertilisers, insecticides, herbicides, fungicides and coagulating agents in rubber industry [1]. Instead of treating TC as a low-value waste stream, coupling this thermochemical platform with microalgal biorefineries offers a sustainable pathway.

Microalgae possess several advantages, including rapid growth rates, efficient CO_2_ fixation, high photosynthetic efficiency, and cultivation independent of arable land (using saline or wastewater resources) [6]. Microalgae-based biorefineries and the use of residual streams as cultivation inputs are widely discussed in the literature. Ho et al. [7] reviewed the torrefaction of microalgal biomass for solid biofuel production, whereas Melikoglu [8] synthesised recent strategies in which wastes serve as inputs to microalgal biorefineries. While these studies demonstrate the breadth of microalgae–waste integration, the “reverse-coupling” strategy of the use of a lignocellulosic TC as a supplement in microalgal cultivation remains largely unexplored. This integration could improve the overall economics and sustainability of torrefaction, wherein the organic acids and phenolic compounds present in TC are assimilated by stress-tolerant microalgae into valuable products such as polysaccharides, lipids, proteins, and pigments [9,10].

Previous work in this direction has evaluated acetic acid-rich thermochemical liquids with green microalgae such as *Chlamydomonas reinhardtii* and *Chlorella* sp. [11,12]. Yet information is lacking on the response of red microalgae, which are known producers of the valuable metabolite exopolysaccharide (EPS), to the combined organic acids, furans, and phenolics present in TC. The red algal species *Porphyridium* generally demonstrate good tolerance to environmental changes, yet their potential in algal biorefinery remains underexplored. Previous studies were limited on *P. purpureum* for dairy waste water treatment and *P. cruentum* for bioremediation of the water-soluble fraction (WSF) of petroleum fuels and ultra-filtered swine wastewater [13,14,15]. *P. marinum*, a unicellular marine red alga [16,17], has been investigated and recognised for its capacity as a prolific producer of sulphated EPS, making it an ideal candidate for TC valorisation [18,19]. In addition, *P. marinum* is known for industrial applications in the production of metabolites such as the pigment B-phycoerythrin and floridean starch. It also has reported antibacterial, anti-tumour, and anti-biofilm activities [20,21].

The present study hypothesises that TC, despite containing inhibitory organic acids and phenolic compounds, can serve as a sustainable carbon-rich supplement for *P. marinum* at appropriate concentrations, stimulating stress-induced EPS production without severely compromising growth. To verify this hypothesis, *P. marinum* was cultivated in media supplemented with different concentrations of TC, and the effects on microalgal growth, biomass composition, EPS production, biochemical composition and antioxidant activity were evaluated. Emphasis was placed on the amino and fatty acid compositions of the biomass as well as its pigment production to obtain a preview into possible biofuel and nutraceutical applications.

This study evaluated the valorisation of TC using a red microalga and the quantitative and qualitative properties of the resultant EPS and biomass, with potential implications for the development of a novel circular bioeconomy pathway for TC bioconversion using algal biorefineries.

## 2. Results and Discussion

### 2.1. Yield of TC and Characterisation

Torrefaction at 225 °C produced 17 ± 2 mL of TC with a measured pH of 2.7. At this temperature, TC formation was primarily associated with some moisture release and production of low-molecular-weight volatile compounds. Analysis using GC-MS and HPLC revealed acetic acid and formic acid as the major compounds, along with smaller amounts of lactic acid, benzoic acid, HMF, and furfurals. The higher proportion of acetic acid could have resulted from the partial degradation of hemicellulose, whereas limited furfural content suggested limited sugar dehydration at 225 °C [22]. Table 1 depicts the TC composition. Detailed GC-MS and HPLC evaluation data across four temperature conditions are reported in Chandrasekharan et al. [12].

### 2.2. Growth of P. marinum

**Figure 1:**
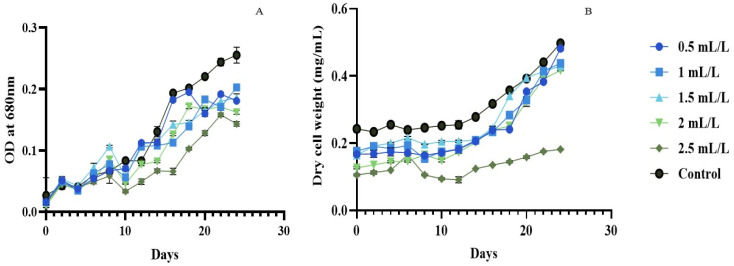
Growth curve of *P. marinum* in different concentrations of TC and in control at OD 680 nm. (**A**) and based on dry cell weight analyses (**B**). TC was added on day 6 at 0.5–2.5 mL/L; untreated cultures served as control.

The growth pattern of *P. marinum* (Figure 1A,B) based on optical density (OD) and dry cell weight (DCW) was studied for about 24 days to analyse the effect of TC on its growth. The OD at 680 nm of the initial cultures was 0.27 ± 0.03, following which *P. marinum* entered the exponential phase with values of 0.5–0.6 by day 6. TC was introduced after day 6 to the culture at concentrations of 0.5–2.5 mL/L. TC induced dose-dependent growth inhibition. At 0.5 mL/L, microalgal growth remained stable, whereas at 1 mL/L, the OD declined from 0.77 ± 0.04 to 0.56 ± 0.04 (day 6 to day 10). At higher concentrations, the growth suppression intensified: 2 mL/L cultures reached an OD of 0.46 ± 0.01 by day 10, while that of 2.5 mL/L dropped to 0.33 ± 0.03. DCW analysis confirmed this pattern, with reductions across all TC concentrations relative to the control.

However, *P. marinum* demonstrated adaptive capacity from day 10 onward. Across the 0.5–2.5 mL/L TC concentration range, the biological response reflected competing substrate and inhibitor effects. At 0.5 mL/L, growth recovered relatively rapidly. Lower concentrations (0.5–1 mL/L) showed recovery within 2–3 days of TC addition, with OD and DCW increasing steadily till day 24. The 1.5 mL/L treatment displayed the strongest mid-range growth, reaching OD 1.14 ± 0.04 by day 14, before stabilising around OD 1.90 ± 0.06 by day 24. Even the 2 mL/L treatment recovered, climbing from OD 0.46 ± 0.01 (day 10) to OD 1.62 ± 0.04 (day 24). The highest concentration (2.5 mL/L) showed the slowest recovery, with OD remaining suppressed at 1.43 ± 0.04 by day 24, roughly 44% below that of the control (2.55 ± 0.13).

After TC addition, the cultures showed an inhibition–recovery response rather than three uniformly resolved growth phases. A definitive stationary phase was not reached within the observation period for the control or the 0.5 mL/L treatment based on DCW. Several treatments showed an apparent late plateau in OD_680_, but DCW continued to change. This divergence may arise because OD_680_ integrates pigment absorption, light scattering and cell concentration [23], whereas DCW represents recovered particulate mass. TC-dependent changes in pigmentation, cell size, aggregation, or EPS could therefore alter the OD_680_-to-DCW relationship without an equivalent change in biomass.

Although direct reports of lignocellulosic inhibitors on *Porphyridium* spp. are limited, the inhibitory effects observed in *P. marinum* are found to align with dose-dependent responses to acetic acid, the major component of TC. Studies on *P. purpureum* show that low sodium acetate concentrations (0.05–0.25%) enhance biomass, while higher concentrations (0.075–1%) inhibit growth [24]. Acetate can support mixotrophic or heterotrophic growth in several microalgae after conversion to acetyl-CoA and entry into the tricarboxylic acid and glyoxylate pathways. Its effect is, nevertheless, concentration- and pH-dependent because the undissociated acid can diffuse into cells, dissociate in the cytosol, and impose proton and anion stress [25]. Furfural, HMF, and phenolic compounds may add oxidative, membrane, and photosynthetic stress, and their combined effect cannot be inferred from acetate alone, suggesting the need for further component-based research.

Low concentrations of xenobiotic or waste-derived compounds can produce biphasic, hormetic responses in microalgae, in which a sub-inhibitory exposure stimulates selected growth or biochemical endpoints while exposure to higher concentrations suppresses them [26]. Such responses have been reported for phenol and other stressors in *Chlorella* species [26,27]. However, the present data do not demonstrate growth hormesis because the endpoint biomass yield did not exceed those of the control.

The recovery observed after day 10 indicates that surviving *P. marinum* cells remained capable of growth under continued TC exposure. Beyond chemical stress, related species such as *P. cruentum* show vulnerability to mechanical and hydrodynamic stress, which are also reported to cause cell damage [28]. Because aeration and vessel conditions were identical among treatments, mechanical and hydrodynamic stress cannot account for the dose-dependent differences observed here.

Research on *P. purpureum* SCS-02 demonstrates that biomass yield corresponds to nitrogen, phosphorus, light intensity, and salinity shifts [29,30]. However, the present measurements do not identify the responsible mechanism. Possible contributors include the utilisation or depletion of readily assimilable compounds, the volatilisation or transformation of inhibitors, physiological acclimation, and changes in medium pH. These alternatives should be tested by time-resolved analysis of TC constituents, pH, cell viability, and photosynthetic performance.

### 2.3. Endpoint Biomass and EPS Yield

The endpoint biomass and corresponding purified EPS yield of *P. marinum* after 24 days are shown in Table 2. At the endpoints, the biomass and EPS yields showed no dose-dependent correlation with TC concentration. The maximum biomass and EPS yield were observed in the control group, whereas these factors were reduced across all TC-treated cultures.

The growth curve data mentioned in the previous section and endpoint EPS yields describe related but distinct features. Several TC-treated cultures resumed growth after the initial decrease, demonstrating survival and partial recovery under continued exposure. However, the endpoint measurements did not show increased biomass or EPS production relative to the control. This pattern may reflect incomplete recovery, treatment-dependent changes in cell composition, or differences in carbon allocation [31], but the present measurements do not establish a particular metabolic mechanism for this phenomenon. Accordingly, the data are interpreted as physiological tolerance and compositional acclimation rather than proof of restored productivity or pathway-specific reallocation [32].

The maximum yield of EPS reported in *P. marinum* was about 33 g in 1056 h in semi-continuous culture whereas in batch culture photoreactors, 18.4 g EPS was produced in 916 h [33].

### 2.4. Biochemical Composition of Biomass

#### 2.4.1. Total Amino Acids and Free Amino Acids

**Figure 2:**
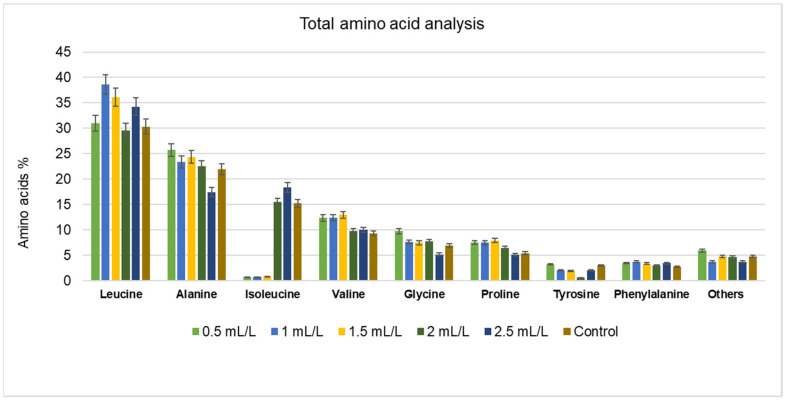
The percentage of total amino acid composition of *P. marinum* cultivated in different concentrations of TC (data are presented as means of three replicates).

Amino acid composition is an important indicator of the nutritional quality of microalgal biomass. To assess the potential suitability of the treated biomass for nutraceutical applications, both total and free amino acid contents were evaluated. The results are reported as relative distributions; therefore, they describe compositional shifts rather than absolute amino acid synthesis. TC exposure altered the relative abundance of several amino acids, including leucine, valine, glycine, and proline; however, endpoint biomass and purified EPS yields remained below the control values. The major total amino acids identified in the microalgal biomass are presented in Figure 2. *P. marinum* contains all essential amino acids; leucine, alanine, isoleucine, valine, glycine, proline, tyrosine, and phenylalanine dominate the profile across all samples, with tryptophan detected only in minor amounts in some TC-supplemented samples.

TC stress shifts the amino acid composition in a concentration-dependent manner, revealing metabolic adaptation. Leucine, valine, and phenylalanine increased in TC-supplemented samples compared to the control. Alanine, glycine, and proline increased until 2 mL/L TC, and declined slightly at 2.5 mL/L, compared to the control. Glycine and proline accumulate strongly under chemical stress and act as osmoprotectants and indicators of abiotic stress responses [34]. Isoleucine showed characteristic drops at 0.5, 1.5, and 2 mL/L TC samples, while valine remained elevated at these concentrations. The inverse relationship between isoleucine and valine reflects feedback inhibition in branched chain amino acid synthesis [35]. In most of the TC-supplemented experiments, an increase was observed in total amino acid content compared to the control, indicating a metabolic shift towards stress-responsive pathways associated with enhanced protein synthesis.

No prior amino acid profiling data exist for *P. marinum*. This study reveals that the species is rich in specific amino acids suitable for targeted applications. In the case of other *Porphyridium* spp. such as *P. cruentum*, all amino acids are present in broader proportions, resulting in a generalised profile [36]. *P. marinum*’s selective enrichment in leucine, alanine, glycine and proline could position it as a candidate for specific amino acid extraction and pathway improvement. Further studies to enhance these pathways could make *P. marinum* a competitive plant-based nutraceutical ingredient.

**Figure 3:**
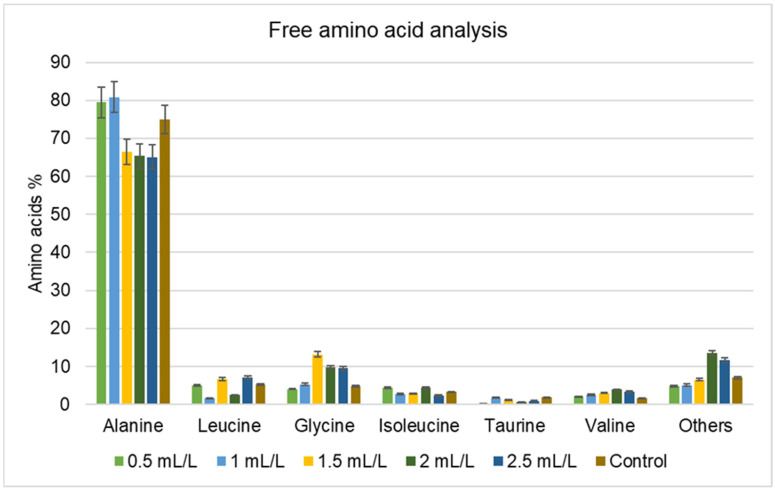
The percentage of free amino acid composition of *P. marinum* cultivated in different concentrations of TC (data are presented as means of three replicates).

Free amino acids are individual amino acids not bound to proteins. Figure 3 shows the free amino acid profile of *P. marinum*. *P. marinum*’s free amino acid profile is dominated by alanine (≥65% across all the samples), with no correlation between amino acid variations and TC concentrations. Beta-alanine and proline (grouped in the category “Others”) were present at 2 and 2.5 mL/L TC concentrations, whereas these were absent in controls and lower doses, indicating abiotic stress response. Proline accumulation is a recognised component of stress acclimation in several eukaryotic microalgae exposed to salinity, osmotic imbalance, metals, and other environmental constraints. Barera and Forlani [37] reviewed the functions of proline in microalgal redox balance, osmoprotection, and stress recovery, and osmotically induced proline accumulation has recently been demonstrated in *Chlorella vulgaris* [38]. In the present study, proline was detected in the high-TC free amino acid profiles but not in the control or lower-dose profiles. This observation is consistent with a possible stress-associated response; however, the data are relative, and the complex TC mixture prevents attribution to acetic acid alone.

Other amino acids such as glycine, leucine, and valine increase across TC-supplemented samples, suggesting the distinct metabolic pathways under stress. Glycine is required for glutathione (GSH) synthesis, and oxidative stress increases GSH demand, which can lead to elevated amounts of glycine [37,39]. In the case of leucine and valine, excess carbon (acetate) leads to branched chain amino acid synthesis [24]. Together, these shifts demonstrate that TC impacts *P. marinum*’s free amino acid profile through multiple interconnected stress pathways. This study reveals a critical gap: amino acid profiles and biosynthetic pathways remain poorly understood in *Porphyridium* species. Further mechanistic studies targeting these pathways could unlock significant industrial and nutraceutical applications of *P. marinum*.

#### 2.4.2. Lipid and Fatty Acid Composition

Total lipid percentages in TC-supplemented and control *P. marinum* lyophilised biomass were evaluated. Lipid percentages decreased with increasing TC supplementation as follows: 47.47% at 0.5 mL/L TC, 37.89% at 1 mL/L, 26.54% at 1.5 mL/L, 19.91% at 2 mL/L, and 18.87% at 2.5 mL/L. Meanwhile, the control biomass showed a lipid content of 15.47%.

**Figure 4:**
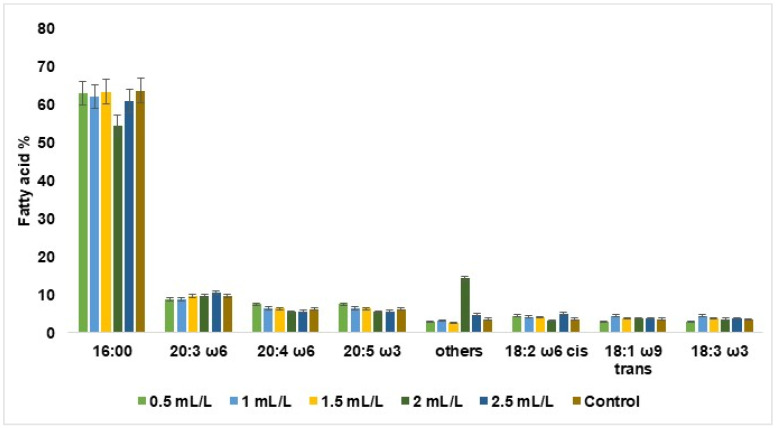
The percentage of fatty acids in the composition of *P. marinum* cultivated in different concentrations of TC. 16:00—palmitic acid, 20:3 ω6—eicosatrienoic acid, 20:4 ω6—arachidonic acid, 20:5 ω3—eicosapentaenoic acid, 18:2 ω6 cis—linoleic acid, 18:1ω9 trans-elaidic acid, 18:3 ω3—the sum of oleic acid, linoelaidic acid, linolenic acid (which is not separated by the GC method) and others, i.e., all other minor detected fatty acids (data are presented as means of three replicates).

The fatty acid analysis (Figure 4) showed that lipid composition shifted towards saturation under stress. Palmitic acid, the dominant fatty acid in the control (63.6%), declined to 54.5% at 2 mL/L TC, reflecting a stress threshold, and then recovered at a higher concentration (2.5). The polyunsaturated fatty acids, arachidonic acid and eicosapentaenoic acid, declined progressively with TC concentration, suggesting a shift toward lipid saturation under chemical stress. This indicates a shift toward membrane stabilisation under oxidative pressure. This compositional reallocation may represent a stress-adaptive strategy to reduce membrane permeability and maintain cellular integrity [40].

The total lipid percentage of the *Porphyridium* spp. varies widely depending upon the cultivation strategy, light intensity, and nutrient limitation. Cultivation of *P. cruentum* on agro-industrial by-products showed a variation in lipid percentage from 11.34% to 24.75% according to the concentrations of beet molasses, corn steep liquor, and f/2 nutrients [41]. Another media optimisation study on *P. purpureum* also showed a wide range of variations from 3.1% to 19.44% due to changes in the experimental conditions [42]. In a novel two-phase cultivation strategy to enhance lipid accumulation in *P. purpureum*, an improved yield of 26.73% lipid content was reported. Here, nitrogen deprivation, red light, high irradiance, melatonin, and nanoparticles jointly enhanced the productivity [43]. When comparing our results to the available literature on *Porphyridium* spp., variations in lipid content appear to be a common occurrence in this species.

All TC-treated biomass showed a numerically higher lipid fraction than the control (15.47%), with the maximum at 0.5 mL/L (47.47%) and a progressive decline to 18.87% at 2.5 mL/L. This pattern coincided with immediate growth inhibition followed by partial recovery, endpoint biomass and EPS yields that did not exceed the control, and shifts in relative amino acid composition. Thus, TC exposure affected biomass composition more strongly than it improved biomass accumulation. Concurrent with this decrease, fatty acid composition shifted toward saturation, with palmitic acid declining at the critical 2 mL/L threshold while polyunsaturated fatty acids declined progressively. These dual responses of reduced total lipid content and increased saturated fatty acid dominance suggest that *P. marinum* reallocates its lipid reserves to fuel energetically demanding stress adaptation pathways, including the enhanced protein synthesis observed in the EPS and amino acid profiles. Thus, TC exposure appears to affect biomass composition more strongly than it improves biomass accumulation.

#### 2.4.3. B-Phycoerythrin (PE) Content

PE content in lyophilised *P. marinum* biomass ranged from 0.75% to 0.96% across TC concentrations, compared to 0.88% in the control. Previous reports on PE content in *Porphyridium* sp. range between 3 and 8% under optimal culture conditions [44], suggesting that the values observed in this study were comparatively lower. The observed differences in PE percentage across TC concentrations (0.75% to 0.96%) did not differ significantly. This suggests that TC concentration had minimal direct influence on PE biosynthesis. PE production is known to be influenced by various environmental factors. Elevated nitrogen and moderate light enhance PE accumulation, whereas stress and high light conditions promote EPS production [45]. Additionally, the presence of phosphorus content and trace metals are also reported to influence PE biosynthesis [44]. Relatively low PE content was observed in all samples of this study, which could reflect pigment loss during the biomass washing step rather than a direct inhibitory effect of TC on PE synthesis.

### 2.5. Structural Characterisation of EPS

#### 2.5.1. Molecular Weight of EPS

Size exclusion chromatography was used for evaluating the weight-average molecular weight (Mw) and the polydispersity index (PDI) of EPS. The control and 1 mL/L TC-treated samples displayed similar molecular weights of about 11,860 kDa and 11,800 kDa, with PDI values of 3.7 and 2.7, respectively. The 2.5 mL/L TC-treated sample displayed a higher Mw of 12,000 kDa with a considerably higher PDI of 4.2. While the 0.5 and 2 mL/L TC treatments resulted in the highest respective molecular weights (14,000 kDa and 13,120 kDa), their respective PDIs were lower (1.8 and 3.1). The lowest Mw (11,200 kDa) with a PDI of 4.0 was observed for the TC treatment concentration of 1.5 mL/L. The high PDI values across most samples reflect a broad molecular weight distribution within the EPS fractions.

Previously reported molecular weights for EPS from *Porphyridium* spp. range between 2 and 7 × 10^6^ Da, and they mainly contain glucuronic acids and sulphate groups [46]. Various factors influencing EPS molecular weight are reported, including culture conditions, extraction methods, and sulphation degree [47,48]. Recent studies on *P. purpureum* showed that nitrogen availability significantly affects the Mw of EPS [30]. The *P. marinum* EPS reported by Soanen et al. (2016) [33] had an Mw of 1.8 × 10^6^ Da with a PDI of 1.1, whilst another study reported an Mw of 1400 kDa (equivalent to 1.4 × 10^6^ Da) with a calculated PDI of 1.5 [21]. The comparatively higher Mw values observed in this study may be associated with the specific culture conditions, extraction methodology, or inherent heteropolysaccharide composition of the EPS. The EPS of *P. marinum* comprises a heteropolysaccharide matrix containing glucose, xylose, galactose, and uronic acid residues [33], combined with sulphate groups [48]. These structural features may facilitate intermolecular interactions and the formation of an aggregated or network-like structure. Additionally, the extraction and purification methods themselves can influence the molecular weight and polydispersity observed during SEC analysis.

#### 2.5.2. FTIR Analysis

**Figure 5:**
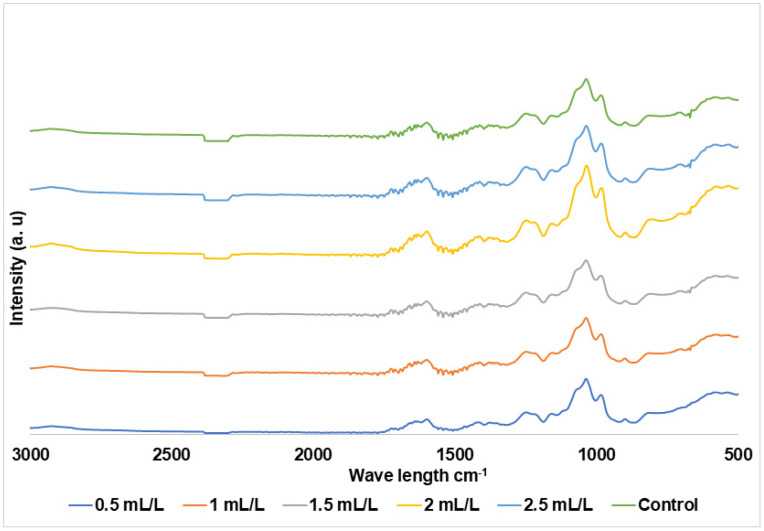
FTIR spectra of EPS from *P. marinum* in different concentrations of TC in medium and control.

The EPS of TC-treated *P. marinum* resulted in FTIR spectra which were not characteristically different from the control samples, as shown in Figure 5, indicating that the functional groups of EPS remained mostly unaffected by TC treatment. This finding aligns with previous findings reported for *C. reinhardtii* [12]. The observed spectral bands were similar with previously reported for *P. marinum* EPS [21], which confirms the integrity of the polysaccharide structure across all treatments.

The 3286–3371 cm^−1^ absorption band was attributed to the O–H stretching vibrations, a characteristic feature of polysaccharides, which is widened by hydrogen bonding and the presence of bound water molecules [49]. The bands detected at 2923–2925 cm^−1^ correspond to aliphatic C–H stretching from CH and CH_2_ groups, commonly associated with carbohydrate backbone structures [50]. The carbonyl peak at 1710–1724 cm^−1^ arises from esterified or acetylated uronic acid residues, confirming the presence of uronic acids in the EPS, a finding supported by the biochemical quantification presented in the following section. The asymmetric carboxyl vibrations at 1631–1414 cm^−1^ further corroborate the presence of uronic acids. The characteristic sulphate ester bands at 1379–1217 cm^−1^ align with previously reported values for *P. marinum* EPS (1222 cm^−1^), indicating the preservation of sulphated extracellular polysaccharides [21]. Glycosidic C–O–C and the C–O stretching displayed at 1150–1038 cm^−1^ correspond to a region mostly reported in carbohydrates. Finally, the deformation of β–C1 anomeric bonds appeared at 978–897 cm^−1^ [21].

The common band positions indicate retention of the principal sulphated polysaccharide functional groups across treatments. This qualitative similarity aligns with the biochemical analyses, which showed only modest variation in uronic acid, protein, and sulphate percentages, and with SEC, which indicated treatment-dependent differences in apparent molecular-weight distribution rather than loss of the polysaccharide backbone. FTIR therefore supports preservation of the major functional group classes but does not, by itself, demonstrate an identical fine structure or explain the assay-dependent antioxidant activity.

### 2.6. Biochemical Composition and Antioxidant Capacity of EPS

#### 2.6.1. Biochemical Composition of EPS

The results of the different biochemical evaluations of EPS from *P. marinum* are given in Table 3. The total sugar, neutral sugars, and acidic sugar values remained closer to the control in all of the samples. However, TC influenced protein content across all concentrations, with maximum protein at 2.5 mL/L (2.89%). Sulphate content showed a distinct pattern, peaking at 9.56% and 9.09% for 0.5 mL/L and 1 mL/L, respectively, suggesting a non-linear dose response.

In *Porphyridium* spp., widely observed ranges are 50–80% total sugars, 40–60% neutral sugars, 10–20% uronic acids, 2–10% proteins, and 5–15% sulphates [51,52,53]. Here, the results vary from the reported ranges. This could be due to culture conditions, strain variability, analytical methods, and purification processes. Previously, similar variations were reported in *P. purpureum*, *P. sordidum*, and *P. cruentum* [51,53]. Despite these variations, the consistent pattern across the data demonstrates that TC stress specifically alters protein and sulphate metabolism in *P. marinum* EPS, indicating a targeted biochemical response to chemical stress [54].

#### 2.6.2. Antioxidant Activity of EPS

**Figure 6:**
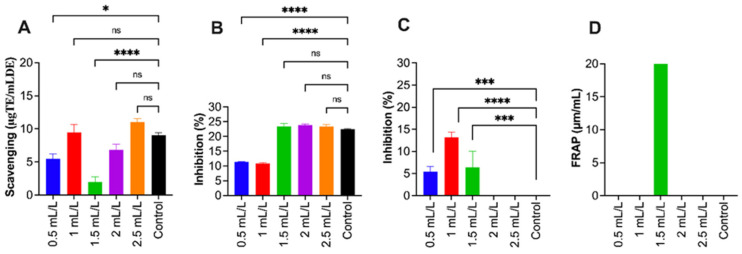
The antioxidant activity of EPS from *P. marinum*. (**A**) ABTS, (**B**) SOD, (**C**) OH radical assay, and (**D**) FRAP. All experimental data are expressed as the mean ± standard error of the mean (*n* = 8); the data are compared to the values of the control group. (*) indicates a statistically significant difference using ANOVA, followed by Dunnett’s multiple comparisons test: **** *p* < 0.0001, *** *p* < 0.001, * *p* < 0.05 and ns—not significant (*p* > 0.05).

EPS from *P. marinum* was analysed for its antioxidant potential using five assays: DPPH, ABTS, SOD-like activity, OH radical scavenging, and FRAP. Overall, EPS revealed weak antioxidant activity across most assays, with concentration-dependent and assay-specific variations, as shown in Figure 6.

DPPH and FRAP assays showed negligible activity. The EPS displayed minimal DPPH radical scavenging across all tested concentrations. These results are analogous to the antioxidant activities of EPS-C from *P. cruentum* (CCALA 415) and EPS-P of *P. purpureum* FACHB 806, where the antioxidant properties of EPS are affected by multiple factors including protein, uronic acids, and phenolic or other redox-active components [55]. Similarly, significant ferric reducing activity was observed at 1.5 mL/L (25.61 ± 36.81, *p* < 0.0001), whereas other concentrations did not differ significantly from the control group. The results show that the electron-donating capacity of the EPS is limited and changes with concentration.

SOD-like activity and OH radical scavenging displayed concentration-dependent responses for moderate doses. EPS enhanced SOD-like activity at lower concentrations (0.5–1.5 mL/L: 11.33–23.46%), reaching a plateau at 2–2.5 mL/L (23.85–23.31%, compared to the control 22.43 ± 0.19%). The EPS exhibited significant OH radical scavenging at 0.5 mL/L (5.39 ± 1.24%, *p* = 0.001), 1 mL/L (13.23 ± 1.19%, *p* < 0.0001), and 1.5 mL/L (6.37 ± 3.70%, *p* = 0.0001), but higher concentrations showed no significant difference from the control. These results display that the EPS can scavenge OH radicals at moderate concentrations; however, the activity results show a non-linear response.

ABTS radical scavenging of EPS showed limited yet detectable activity. Significant differences appeared at 0.5 mL/L (5.48 ± 0.72%, *p* = 0.0188) and 1.5 mL/L (1.95 ± 0.80%, *p* < 0.0001), while other concentrations (1, 2, 2.5 mL/L) were not significantly different from the control (9.02 ± 0.43%). This trend varies markedly from other reported results of *P. cruentum* (35.97% ABTS activity at 5 mg/mL) and *P. purpureum* (47.02% scavenging activity), suggesting that strain variability and cultural conditions can influence antioxidant capacity [55].

Previous studies have reported higher sulphate content in polysaccharides, resulting in higher antioxidant activity [54]. However, all samples in this study had more than 8% sulphate content and still demonstrated comparatively lower antioxidant activities. This suggests that sulphate alone may not be the determining factor of antioxidant potential in *P. marinum* EPS. The functional groups present in EPS seem to contribute differently to various antioxidant mechanisms, in which certain groups display activity against specific radicals (OH, SOD), whereas others (DPPH, FRAP) remain non-reactive. Thus, future research could focus more on enhancing biological activity through approaches such as chemical modification, enzymatic treatment, or conjugation with bioactive molecules [56].

## 3. Materials and Methods

### 3.1. Biomass Torrefaction and Characterisation of TC

Torrefaction of 200 g of aspen wood chips was conducted in a batch reactor at 225 °C for a residence time of 1 h. To maintain an oxygen-free environment, nitrogen was purged at a 5 L/min flow rate. Volatile compounds were captured using a condenser (water-cooled) into a glass bottle attached to the reactor. The collected TC was measured and recorded. TC was characterised by GC-MS (Agilent 7890B, (Agilent Technologies, Santa Clara, CA, USA)) and HPLC (Shimadzu, Kyoto, Japan), following Chandrasekharan Nair et al. [12].

### 3.2. Cultivation of Microalgae

The microalgal strain *P. marinum* (CCAP 1380/10) was purchased from the Culture Collection of Algae and Protozoa, Dunbeg, Scotland, United Kingdom (UK) (https://www.ccap.ac.uk/catalogue/strain-1380-10, accessed on 16 February 2022). Cultures were grown and maintained in modified PM medium containing (g/L): CaCl_2_·2H_2_O (1.55), MgSO_4_·7H_2_O (7.2), NaNO_3_ (1.7), NaCl (15), K_2_HPO_4_ (0.9), and 1 mL/L trace metal solution comprising MnSO_4_·H_2_O (0.15 g/L), (NH_4_)6Mo_7_O_24_·4H_2_O (0.015 g/L), CoSO_4_·7H_2_O (0.015 g/L), CuSO_4_·5H_2_O (0.05 g/L), ZnSO_4_·7H_2_O (0.25 g/L), and Fe-EDTA (10 g/L). Vitamin stock (1 mL/L) was prepared separately in Milli-Q water (Elix water purification system, Millipore, Merck KGaA, Darmstadt, Germany) and contained vitamin B12 (0.01 g/L), thiamine (0.025 g/L), and biotin (0.04 g/L) (Soanen et al., 2016) [33]. The first five salts in medium were autoclaved for 15 min at 121 °C. Trace element and vitamin solutions were sterilised by filtration through 0.2 µm filters and aseptically added post autoclaving. Cultures were maintained at 23–25 °C under continuous illumination with an irradiance intensity of 216 µmol photons·/m^2^/s and a 24 h photoperiod.

Experiments were carried out in 500 mL Schott glass bottles with 450 mL medium. Starter cultures which were maintained per culture collection protocols were inoculated at 10% (*v*/*v*) with an optical density (OD_680_) of ≈0.2–0.3. Continuous artificial illumination (216 µmol photons/m^2^/s^1^) and aeration (flow rate 0.5 mL/min) were provided. Cultures were maintained at 23–25 °C, where OD at 680 nm (OD_680_) was monitored at 48 h intervals and evaporative losses were compensated by the addition of fresh medium. After 6 days, when the cultures’ OD_680_ reached 0.1–0.2, TC was introduced at final concentrations of 0.5, 1, 1.5, 2, and 2.5 mL/L. L The concentration of TC was decided based on the preliminary results. Cultures were grown for 18 days post TC addition, and medium pH was monitored. Cell growth was assessed by OD_680_ measurement using a UV-Vis spectrophotometer along with DCW analysis. For DCW determination, 10 mL of microalgal culture was collected onto Whatman filter paper (pre-dried and pre-weighed) by vacuum filtration, and cells were dried at 120 °C overnight, until a constant weight was achieved. All experiments were performed in triplicate, with cultures grown in medium without TC as the control.

### 3.3. Harvesting Algal Biomass and EPS Extraction

The microalgal culture was centrifuged for 5 min at 10,000 rpm at 20 °C to harvest the algal biomass as pellets separated from the medium. The pellets were washed with dilute medium to remove excess salts, then freeze-dried for storage at −20 °C for various analyses [13]. EPS was precipitated from the supernatant using 98% ethanol (ice cold) at a 1:4 ratio, followed by overnight incubation at 4 °C. After overnight incubation, centrifugation was performed for 3 min at 10,000 rpm (4 °C) to recover EPS from an ethanol–medium mixture. The extracted EPS was dispersed in Milli-Q water. The EPS was further purified with an Amicon filter cell (Merck KGaA, Darmstadt, Germany) fitted with a 10 kDa MWCO polyethersulphone membrane (Biomax^®^ Ultrafiltration discs, Millipore, Burlington, MA, USA). Purification continued till the sample’s conductivity reached 0.2 mS/m. The purified samples were then freeze-dried for further analysis [57].

### 3.4. Biochemical Analyses of P. marinum Biomass

#### 3.4.1. Amino Acid and Protein Content Analyses

The amino acids, both free and total, in freeze-dried *P. marinum* biomass were analysed using GC-MS. For total amino acids analysis, a 0.02 g sample was hydrolysed for 15 h at 120 °C with 2 mL of 6 M HCl. Hydrolysed samples were dried at 95 °C (nitrogen atmosphere) and dissolved in Milli-Q water (2 mL) for GC-MS analysis. For analysis of free amino acids, 0.05 g of biomass was mixed with 0.1 M HCl (1.5 mL), vortexed at 1400 rpm at room temperature for 5 min, and centrifuged for 15 min at 21,000× *g* at 4 °C. The resulting supernatant was collected and kept at −80 °C. Both total and free amino acids followed identical preparation steps for GC-MS analysis.

Amino acid samples (100 µL) were combined with acetonitrile (250 µL) and centrifuged for 3 min at 21,000× *g*. The supernatant (100 µL) was mixed with a DL-norleucine internal standard (100 µL of a 5 µg/mL) in a heat-resistant screw cap microcentrifuge tube (Thermo Scientific™, Bremen, Germany). Samples evaporated under nitrogen were mixed with dichloromethane (50 µL) and evaporated again. After evaporating samples were combined with MTBSTFA (100 µL SUPELCO, 77,626, Bellefonte, PA, USA) and acetonitrile (100 µL), then incubated at for 1 h at 100 °C for derivatisation. Post incubation, the tubes were centrifuged for 15 min at 21,000× *g* at 4 °C, and 200 µL of supernatant was collected in a vial, which again was centrifuged for 5 min at 2000 rpm before GC-MS analysis.

The ultra gas chromatograph system Shimadzu GCMS-QP2010 (Kyoto, Japan), equipped with a mass spectrometry (MS) detector, was used for amino acid analysis. Separation was achieved using a silicon-filled capillary column—Phenomenex Zebron ZB-5MS (Torrance, CA, USA) (30 m × 0.25 mm, 0.25 μm layer thickness) with helium gas (flow rate of 1 mL/min). The MS detector was retained at 325 °C, and the ion source was maintained at 300 °C. The sample injector, with a 2 mm diameter straight liner, operated at 280 °C with a 0.5 µL injection volume in split mode (distribution flow 100), scanning from *m*/*z* = 25 to 500. During analysis, the column was held for 2 min at 100 °C, then heated to 298 °C at rate of 5 °C/min, and maintained for 25 min. For quantification, analytical standards (Sigma-Aldrich, AAS18, Saint Louis, MO, USA) were utilised [58].

#### 3.4.2. Lipid Extraction and FAME Analysis

Lipid extraction from lyophilised *P. marinum* biomass was performed using a chloroform/methanol (2:1, *v*/*v*) solvent system. About 0.3 g of biomass was weighed in a glass screw-capped tube and combined with methanol (1 mL) and chloroform (2 mL), followed by vigorous mixing for 90 s. This was followed by incubation for 30 min at 40 °C in a water bath. Following incubation, 2% NaCl solution (1.25 mL) and 1.25 mL chloroform were added and mixed thoroughly, and centrifuged for 20 min at 1700× *g*. Using a glass Pasteur pipette, the chloroform layer was transferred into pre-weighed glass tubes, dried under nitrogen gas, and re-weighed to determine the total lipid content expressed in percentage dry weight.

For conducting fatty acid methyl ester (FAME) analysis of the sample, 5% sulfuric acid (1.5 mL) in methanol was added to the dried samples. The mixture was then incubated at 50 °C for 1 h, with intermittent mixing for 30 s every 15 min. After cooling in an ice water bath, 1 mL of Milli-Q water and 1.5 mL of hexane were added to the mixture followed by mixing and phase separation. The upper layer was collected in HPLC vials followed by drying under nitrogen. Further, hexane (500 µL) was added to the samples and kept at −20 °C for GC analysis.

A Shimadzu GCMS-QP2010 Ultra system with a mass spectrometric detector was used for GC-MS analysis. The analytical settings used were identical to those described previously for amino acid analysis. The temperature of the column increased to 260 °C from 160 °C with a heating rate of 2.5 °C/min, further to 298 °C with a heating rate of 5 °C/min, and was maintained for 15 min. For fatty acid identification, 37 FAME Mix (Supelco CRM47885, Sigma-Aldrich, Taufkirchen, Germany) was used as reference/calibration standards [58].

#### 3.4.3. PE Extraction and Quantification

**Figure 7:**
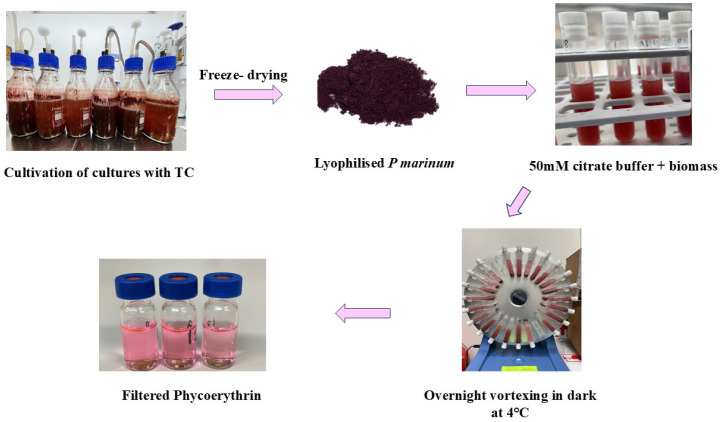
Representation of phycoerythrin extraction from *P. marinum*.

PE was extracted (Figure 7) from lyophilised *P. marinum* biomass using 50 mM citrate buffer (pH 6) prepared with Na_3_C_6_H_5_O_7_, 2H_2_O and NaC_6_H_7_O_7_. Biomass samples weighed (approximately 0.017–0.022 g) in a plastic tube suitable for IKA Looper were mixed with citrate buffer by weighing to make a final suspension of 0.025% (*w*/*w*). A small metal ball (d~3mm) was introduced to each tube, followed by stirring of the mixture. The sample solution was placed in Looper for 35 rpm for 24 h in dark at 20 °C with stirring. Following incubation, centrifugation of the tubes was carried out for 15 min at 2000× *g* and then the samples were filtered using a 0.45 μm RC syringe membrane (Sartorius, Göttingen, Germany) in HPLC vials for HPLC analysis and Eppendorf tubes for absorption spectrum measurements with a spectrophotometer operating under baseline correction. The filtered samples were transferred into vials and analysed using a Shimadzu HPLC (Shimadzu, Kyoto, Japan) platform equipped with a Nexera X2 LC-30AD pump and a Nexera X2 SIL-30AC autosampler (Shimadzu, Kyoto, Japan). Column temperature was maintained using a Shimadzu CTO-20AC oven (Shimadzu, Kyoto, Japan). Detection was carried out with a Prominence RF-20A fluorescence detector (Shimadzu, Kyoto, Japan) operating at excitation and emission wavelengths of 542 and 575 nm, respectively, together with a Prominence SPD-M20A PDA detector (Shimadzu, Kyoto, Japan) monitored at 280 and 542 nm. Phenomenex columns, OHpak SB-804 HQ (Shodex, Tokyo, Japan), were used in series for the HPLC analysis. The mobile phase was Na-phosphate (50 mM) and NaCl (300 mM, pH 6.5) with a flow rate of 0.7 mL/min. The autosampler was maintained at 10 °C and oven temperature was maintained at 20 °C. Sample injection volumes of 5 µL and 100 µL were used for the fluorescence detector and PDA detector, respectively [59].

### 3.5. Structural Characterisation of EPS

#### 3.5.1. Size Exclusion Chromatography of EPS

EPS was prepared at a concentration of 0.5 mg/mL using 0.1 M NaNO_3_ solution. Complete dissolution was achieved by vigorous stirring in a boiling water bath. After heating, the samples were maintained at approximately 60 °C and passed through a 0.22 μm cellulose acetate syringe filter (25 mm). The filtrates were collected in HPLC vials and allowed to cool to 40 °C prior to analysis. Size exclusion chromatographic analysis was performed using a Shimadzu HPLC system equipped with a Nexera X2 LC-30AD unit and a CBM-20A controller (Shimadzu, Kyoto, Japan). Chromatographic separation was achieved using two OHpak SB-806MHQ columns (300 × 8 mm) (Shodex, Tokyo, Japan) connected in series along with an OHpak SB-G guard column (Shodex, Tokyo, Japan). A 0.1 M NaNO_3_ solution served as the mobile phase with a flow rate of 0.8 mL/min, while the column compartment temperature was maintained at 60 °C. Sample aliquots of 100 µL were introduced through a Nexera X2 SIL-30 AC autosampler (Shimadzu, Kyoto, Japan) operated at 40 °C. Each analysis was performed with a total run time of 45 min. Detection was carried out using an RID-10A refractive index detector (Shimadzu, Kyoto, Japan) coupled with the LabSolutions data acquisition system. Molecular weight estimation of the samples was based on a calibration curve generated from 12 pullulan standards ranging between 0.342 and 2400 kDa, with data processing conducted using LabSolutions software version 5.97 (Shimadzu, Kyoto, Japan) [58].

#### 3.5.2. FTIR Spectroscopy of EPS

Purified lyophilised polysaccharide samples were analysed for chemical composition using FTIR (iS50) Nicolet (Thermo Fisher Scientific, Waltham, MA, USA). The EPS sample was placed on ATR diamond crystal using cleaned and dried forceps. At room temperature, IR spectra were measured in absorption mode within the 400–4000 cm^−1^ range, followed by data processing using Omnic software version 9.2. 

### 3.6. Biochemical Analysis of EPS

Standard analyses were conducted in duplicate, whereas all sample analyses were carried out in triplicate.

#### 3.6.1. Total Carbohydrate Determination

The Dubois phenol–sulfuric acid procedure was employed for determination of total carbohydrate concentration. EPS solutions were prepared in Milli-Q water at a concentration of 1 mg/mL. Subsequently, 500 μL of the EPS solution was transferred into a screw-capped glass tube and combined with an equal volume of phenol solution (50 g/L). Immediately afterward, 2.5 mL of concentrated H_2_SO_4_ was introduced, and the reaction mixture was allowed to stand at room temperature for 10 min before vortex-mixing at 3000 rpm for 10 s. The mixture was then further incubated at room temperature for 15 min, followed by heating in a 35 °C water bath for 30 min. Absorbance readings were recorded at 483 nm using a V-630 UV–Vis spectrophotometer (JASCO Corporation, Hachioji, Tokyo, Japan). A calibration curve was generated with a 0.1 g/L glucose standard solution, while Milli-Q water served as the blank [60].

#### 3.6.2. Neutral Sugar and Uronic Acid Determination

Neutral sugar and uronic acid contents of the purified EPS samples were determined using the resorcinol and meta-hydroxyldiphenyl (m-HDP) assays, respectively. Glucose and glucuronic acid were employed as calibration standards. For neutral sugar analysis, 200 μL of EPS solution (1 mg/mL) was transferred into a screw-cap glass tube and mixed with 200 μL resorcinol reagent together with 1 mL of 80% H_2_SO_4_. After vortex mixing, the reaction tubes were heated in a 90 °C water bath for 30 min and subsequently cooled to room temperature in the dark. The reacted mixture was then diluted with 1.4 mL Milli-Q water, vortexed again, and absorbance was recorded at 450 nm using a UV–Vis spectrophotometer (V-630 JASCO Corporation, Hachioji, Tokyo, Japan). The neutral sugar content was estimated and expressed as D-glucose equivalent in mg/g (GlcEq) [61].

For uronic acid estimation, 200 μL of 1 mg/mL EPS solution was mixed with 1 mL of a borax solution (sodium tetraborate 0.12 M, in conc. H_2_SO_4_) in a screw-capped glass tube, vortexed, and incubated in a boiling water bath for 1 h at 90 °C. Subsequent to incubation, the samples were treated with 200 μL freshly prepared m-HDP solution (100 mg/mL in DMSO) and vortexed thoroughly. The mixtures were then reheated at 90 °C for 2 min, after which absorbance was determined at 520 nm using a UV–Vis spectrophotometer (V-630 JASCO Corporation, Hachioji, Tokyo, Japan). The results were reported as mg/g D-glucuronic acid equivalents (GlcA Eq) [62].

#### 3.6.3. Protein Determination

Protein content of the EPS samples was quantified using the Bradford assay based on the Coomassie Brilliant Blue G-250 method. Bovine serum albumin (BSA) served as the calibration standard, while Milli-Q water was employed as the blank control. For the analysis, 200 μL of Bradford reagent (Bio-Rad Laboratories, Hercules, CA, USA) was combined with 200 μL of EPS solution (1 mg/mL) and 600 μL of Milli-Q water. After vortex-mixing, the reaction mixture was incubated at room temperature for 10–60 min, and absorbance was subsequently recorded at 595 nm using a spectrophotometer. (V-630 (JASCO Corporation, Hachioji, Tokyo, Japan)) [63].

#### 3.6.4. Sulphate Determination

The degree of sulphation in polysaccharides was quantified using a modified turbidimetric assay with BaCl_2_/gelatin and K_2_SO_4_ as the calibration standard. A gelatin solution was prepared by dissolving 150 mg of gelatin in 50 mL of Milli-Q water at a temperature of 70 °C, followed by cooling at 4 °C for 16 h, and then adding 0.5 g BaCl_2_·2H_2_O and homogenising to obtain the BaCl_2_/gelatin reagent. For hydrolysis, 20 mg of polysaccharide was mixed with 1.0 mL of HCl (2 M) in a glass tube and then heated for 2 h at 100 °C. The resulting hydrolysate was further centrifuged for 30 min at 13,000× *g* at 20 °C, and the supernatant was collected. The assay mixture consisted of 0.5 mL supernatant, 0.5 mL hydrochloric acid (0.5 M), and 0.25 mL BaCl_2_/gelatin reagent. After vortexing and incubation for 30 min at room temperature, turbidity was measured spectrophotometrically at 550 nm following brief mixing (JASCO Corporation, Hachioji, Tokyo, Japan) [53,64].

### 3.7. Evaluating Antioxidant Activity of EPS

#### 3.7.1. DPPH Assay

The radical scavenging activity of the purified EPS was assessed using DPPH (2,2-diphenyl-1-(2,4,6-trinitrophenyl) hydrazin-1-yl) via the Brand–Williams method with modifications by Miliauskas et al. [65,66]. An aliquot of 20 μL of 0.1% EPS solution was combined with 180 μL of 0.1 mM DPPH prepared in absolute ethanol and incubated in the dark at room temperature for 30 min. Trolox (1 mM in absolute ethanol) was used as the reference standard, while Milli-Q water served as the blank control. Following incubation, absorbance was measured at 570 nm using an OPTIMA microplate reader (BMG LABTECH, Ortenberg, Germany).

#### 3.7.2. ABTS Assay

A working solution of ABTS (containing 3.5 mM ABTS with 1.225 mM Potassium persulphate) was prepared. Subsequently, 120 μL of the prepared solution was combined with 30 μL of 0.1% EPS solution and incubated in the dark for 30 min at 37 °C. Trolox and ascorbic acid were used as reference standards, whereas Milli-Q water served as the blank control. After incubation, the absorbance of the reaction mixture was measured at 734 nm using a microplate reader [67].

#### 3.7.3. Superoxide Dismutase (SOD) Assay

The SOD scavenging activity of the purified EPS solution (0.1% *w*/*v*) was determined using a modified protocol adapted from Li (2012) [68]. About 12 µL of EPS solution was added to 180 µL of Tris–HCl buffer (50 mM, pH 8) containing EDTA (10 mM), followed by the addition of 2 µL of pyrogallol (60 mM). The reaction mixture was incubated for 5 min at 37 °C, and absorbance was subsequently measured using a microplate reader at 325 nm. L-ascorbic acid was used as the positive control, whereas Milli-Q water served as the blank. Superoxide radical scavenging activity was expressed as percentage inhibition using the formula given below:

(1) $$SOD-like\;scavenging\;inhibition\left(\%\right)=\left(\frac{AB-AE}{AB}\right)\times 100$$

*AB* corresponds to the blank’s absorbance, and *AE* corresponds to EPS sample’s absorbance.

#### 3.7.4. Hydroxyl Radical (HO) Assay

For the hydroxyl radical (•OH) scavenging assay, 20 μL of 0.1% EPS solution was mixed with 45 μL of H_2_O_2_ (6 mM), 20 μL of sodium salicylate (20 mM), and 65 μL of FeSO_4_·7H_2_O (1.5 mM). The reaction mixture was incubated for 30 min at room temperature (22–25 °C) in the dark. Following incubation, the absorbance was measured at 562 nm using a microplate reader. Trolox was used as the positive control, while Milli-Q water served as the blank. The OH radical scavenging activity was expressed as percentage inhibition using the corresponding formula.

(2) $$OH\;radical\;scavenging\;capacity\;(\%)=\left(\frac{AB-AE}{AB}\right)\times 100$$

where *AB* is the blank’s absorbance, and *AE* is the sample’s absorbance [67].

#### 3.7.5. FRAP—Ferric Reducing Antioxidant Potential Assay

FRAP reagent was freshly prepared by mixing TPTZ (2,4,6-tri(2-pyridyl)-s-triazine) solution (10 mM) in HCl (40 mM), sodium acetate buffer (300 mM, pH 3.6), and FeCl_3_·6H_2_O (20 mM) at a volume ratio of 10:1:1. (*v*/*v*/*v*). For the assay, 180 μL of the FRAP working reagent was combined with 6 μL of 0.1% EPS solution and incubated in the dark for 30 min at 37 °C. Trolox was used as the reference standard, while acetate buffer served as the blank control. After incubation, the absorbance of the reaction mixture was measured at 593 nm using a microplate reader. The antioxidant capacity was determined using the corresponding calibration equation and expressed as mmol Fe^2+^ equivalents per gram of sample.

(3) $$FRAP\;value=\left(\frac{A_{1}-A_{0}}{A_{C}-A_{0}}\right)\times 2$$

where *A*_1_ is the sample’s absorbance, *A*_0_ is the blank’s absorbance and *A_c_* is the positive control’s absorbance [69].

### 3.8. Statistical Analyses of Antioxidant Data

For the analysis of antioxidant data, all the statistical evaluations were conducted using software GraphPad Prism version 10.0.1 (San Diego, CA, USA), which also facilitated graph preparation. Statistical significance was categorised as **** *p* < 0.0001, *** *p* < 0.001, ** *p* < 0.01, and *p* < 0.05. All experiments were conducted in quadruplicate (*n* = 4). Statistical significance among the experimental groups was evaluated using one-way ANOVA followed by Dunnett’s multiple comparisons test, with significant differences indicated by asterisks (*).

## 4. Conclusions

This study demonstrates that *P. marinum* can survive exposure to low concentrations of aspen wood TC and partially recover after the initial concentration-dependent inhibition. However, the endpoint biomass and purified EPS yields did not exceed the control, and TC should therefore not be described as enhancing growth or EPS production under the tested conditions. The clearest compositional response was the high lipid fraction at 0.5 mL/L (47.47% of dry biomass versus 15.47% in the control), followed by a decrease in lipid percentage as TC dosage increased. Increased lipid fractions could suggest possible biofuel applications. To study the potential of TC as a supplement in microalgal growth for possible food applications, amino acid and fatty acid compositions were studied. Changes were also observed in relative amino acid and fatty acid profiles and in EPS protein and sulphate contents, while FTIR indicated retention of the principal EPS functional groups. As pathway fluxes, intracellular metabolites, and compound uptake were not measured, these patterns are interpreted as compositional acclimation rather than proof of a specific metabolic mechanism. The generally weak and assay-dependent antioxidant activity further limits claims of immediate nutraceutical value.

The results provide a proof of concept for biological tolerance to a dilute torrefaction side stream, not yet an optimised production process. Future work should quantify the individual TC constituents and their fate during cultivation, resolve the relative effects of acidity and specific inhibitors, and examine lower-dose, batch, fed-batch, or detoxified TC addition. Reconciled mass balances and productivities should then be validated in controlled photobioreactors. Application pathways should be assessed separately: lipid-rich biomass may be evaluated for fuel or oleochemical conversion, whereas any food, feed, or nutraceutical use will require contaminant analysis, toxicological assessment, regulatory compliance, and evidence of biological efficacy. Techno-economic analysis and life-cycle assessment will be necessary before scale-up claims can be made.

## Figures and Tables

**Table 1:** Composition of TC at 225 °C.

Composition of TC at 225 °C	
**GC-MS**	**GC-MS % Area**
Carbon dioxide	10.38
Acetic acid	41.86
Furfural	4.53
Benzoic acid, 2,4-bis[(trimethylsilyl)oxy]-, trimethylsilyl ester	5.84
Cyclononasiloxane, octadecamethyl	10.33
Cyclodecasiloxane, eicosamethyl-	15.97
Silane	7.51
2-Thiophenecarboxylic acid hydraz	3.58
**HPLC**	**mg/mL**
Acetic acid	73.40
Formic acid	31.30
Lactic acid	6.00
Furfural	2.37
HMF	0.30

**Table 2:** Mean percentage of endpoint biomass and EPS produced by *P. marinum* at varying TC concentrations.

Sample	0.5 mL/L TC	1 mL/L TC	1.5 mL/L TC	2 mL/L TC	2.5 mL/L TC	Control
Biomass (g/L)	1.44 ± 0.01	1.69 ± 0.04	1.4 ± 0.2	1.51 ± 0.03	1.38 ± 0.04	1.74 ± 0.04
Purified EPS (mg/L)	185.5 ± 0.04	160.4 ±0.04	118.4 ± 0.03	129.9 ± 0	154.8 ± 0.02	202.7 ± 0.22

**Table 3:** Summary of biochemical composition of EPS from *P. marinum*.

Composition of EPS Sample [% Mass (g/100 g EPS)]	Percentage of Total Carbohydrate	Percentage of Neutral Sugars	Percentage of Uronic Acids	Percentage of Proteins	Percentage of Sulphate Groups (%, eq. SO_4_)
0.5 mL/L	90.40 ± 0.03	85.95 ± 0.05	14.05 ± 0.04	2.08 ± 0.06	9.56 ± 0.02
1 mL/L	88.23 ± 0.05	83.68 ± 0.08	16.32 ± 0.07	2.43 ± 0.04	9.09 ± 0.06
1.5 mL/L	87.34 ± 0.06	84.27 ± 0.04	15.73 ± 0.02	2.18 ± 0.03	8.83 ± 0.07
2 mL/L	88.45 ± 0.04	83.66 ± 0.06	16.34 ± 0.07	2.80 ± 0.01	8.54 ± 0.05
2.5 mL/L	89.82 ± 0.09	84.11 ± 0.01	15.89 ± 0.04	2.89 ± 0.07	8.82 ± 0.01
Control	90.94 ± 0.02	85.13 ± 0.07	14.87 ± 0.03	1.67 ± 0.03	8.80 ± 0.04

## Data Availability

The data presented in this study are available upon request from the first author, and the datasets and materials are included within the manuscript.
